# Towards a ground pattern reconstruction of bivalve nervous systems: neurogenesis in the zebra mussel *Dreissena polymorpha*

**DOI:** 10.1007/s13127-017-0356-0

**Published:** 2018-01-18

**Authors:** Anna Pavlicek, Thomas Schwaha, Andreas Wanninger

**Affiliations:** Department of Integrative Zoology, University of Vienna, Althanstrasse 14, 1090 Vienna, Austria

**Keywords:** Mollusca, Bivalvia, Evolution, Neurogenesis, Evodevo, Veliger, Trochophore

## Abstract

Bivalvia is a taxon of aquatic mollusks that includes clams, oysters, mussels, and scallops. Within heterodont bivalves, *Dreissena polymorpha* is a small, mytiliform, freshwater mussel that develops indirectly via a planktotrophic veliger larva. Currently, only a few studies on bivalve neurogenesis are available, impeding the reconstruction of a ground pattern in Bivalvia. In order to inject novel data into this discussion, we describe herein the development of the serotonin-like and α-tubulin-like immunoreactive (lir) neuronal components of *D. polymorpha* from the early trochophore to the late veliger stage. Neurogenesis starts in the early trochophore stage at the apical pole with the appearance of one flask-shaped serotonin-lir cell. When larvae reach the veliger stage, four flask-shaped serotonin-lir cells are present in the apical organ. At the same time, the anlagen of the cerebral ganglia start to form at the base of the apical organ. From the apical organ, one pair of cerebro-visceral connectives projects posteriorly and connects to a posterior larval sensory organ that contains serotonin- and α-tubulin-like flask-shaped cells. Additional, paired serotonin-lir neurites originate from the apical organ and project into the velum. One unpaired stomatogastric serotonin-lir cell develops ventrally to the stomach at the veliger stage. The low number of serotonin-lir cells in the apical organ of bivalve veligers is shared with larvae of basally branching gastropods and scaphopods and is thus considered a feature of the last common ancestor of Conchifera, while the overall simplicity of the larval neural architecture appears to be a specific trait of Bivalvia.

## Introduction

Mollusca is a highly diverse metazoan phylum with its earliest members most likely dating back to the Precambrian (Parkhaev 2008). The remarkable plasticity of bodyplans among molluscan clades, exemplified by worm-shaped forms such as the neomeniomorphs (solenogasters) and chaetodermomorphs (caudofoveates), polyplacophorans, as well as the conchiferan taxa Monoplacophora, Bivalvia, Scaphopoda, Gastropoda and Cephalopoda, renders this taxon particularly well-suited for developmental and evolutionary studies (e.g., Wanninger et al. 2008). Within Mollusca, Bivalvia is the second largest class-level taxon with the majority of species being marine but several linages such as the unionids or dreisseniids have colonized freshwater environments. Bivalves are typically (but not always) bilaterally symmetric with a dorsally hinged bivalved shell that encloses their laterally compressed body. Bivalvia is traditionally considered to contain two major sister clades, Protobranchia and Autobranchia. Bivalves lack several mollusk-specific features such as a buccal mass and radula, as well as salivary and esophageal glands (Giribet 2008). In adult autobranch bivalves, three pairs of ganglia constitute the major part of the nervous system, namely the cerebropleural ganglia (a result of fusion of the cerebral and pleural ganglia), the pedal ganglia, and the visceral ganglia, while some protobranch clades have retained the ancestral condition of distinct cerebral and pleural ganglia (Bullock and Horridge 1965; Wanninger 2016). The molluscan-like tetraneural condition is usually present and includes paired cerebro(pleural)-pedal connectives, which interconnect the cerebral (or cerebropleural) and pedal ganglia, and the cerebro(pleural)- visceral connectives, which emerge from the cerebral (or cerebropleural) ganglia and extend posteriorly to connect to the visceral ganglia (Wanninger 2016).

Most bivalves are dioecious and release their gametes into the water column, where external fertilization takes place (e.g., Zardus and Martel 2001). Apart from this, brooding of the larvae in the mantle cavity is also known in species that are small and/or, in particular, freshwater and deep water species, especially those that burrow in soft or hard substrates (Zardus and Martel 2001; Cragg et al. 2009). Most bivalves develop via an initial trochophore larva which is followed by a veliger stage that already shows a distinct foot anlage (Cragg 1996; Giribet 2008). The pericalymma larva of protobranch bivalves as well as the parasitic glochidium larva of freshwater unionids provide exceptions to this common developmental type (e.g., Cragg 1996; Zardus and Morse 1998; see also Giribet 2008 for summary). The zebra mussel *Dreissena polymorpha* (Pallas 1771), a small, mytiliform freshwater bivalve, exhibits the common bivalve life cycle involving external fertilization and development through trochophore- and veliger-type larvae. Settlement is followed by gradual metamorphosis into the juvenile whereby individuals reversibly attach to various hard substrates by proteinous byssus threads (Eckroat et al. 1993; Morton 1993; Ram et al. 1996; Taylor et al. 2007; González et al. 2015).

Although several classical accounts on bivalve development and organogenesis exist, these had been limited to light microscopical investigations for many decades (e.g., Hatschek 1880; Meisenheimer 1899, 1901). A number of electron microscopical studies followed that largely focussed on gross morphological larval features (e.g., Cragg 1996; Marois and Carew 1997a, b; Zardus and Morse 1998). With routine establishment of techniques such as immunofluorescence labeling and confocal microscopy, detailed studies on morphogenesis such as neuromuscular development of minute specimens including bivalves are now possible (e.g., Wurzinger-Mayer et al. 2014). However, while numerous invertebrate taxa have been treated in detail, bivalves have so far been largely neglected. As such, within Mollusca, a solid database on neurogenesis exists for the gastropods (e.g., Croll and Voronezhskaya 1996; Dickinson et al. 2000; Dickinson and Croll 2003; Croll 2006; Wollesen et al. 2007; Page and Kempf 2009; Kristof and Klussmann-Kolb 2010; Kristof et al. 2016), polyplacophorans (Friedrich et al. 2002; Haszprunar et al. 2002; Voronezhskaya et al. 2002), and, to a far lesser degree, for aplacophorans (Redl et al. 2014), scaphopods (Wanninger and Haszprunar 2003), cephalopods (Wollesen et al. 2008, 2010) and bivalves (Kreiling et al. 2001; Voronezhskaya et al. 2008).

Apart from the four longitudinal connectives, molluscan larvae typically exhibit an apical sensory organ as a shared feature with other lophotrochozoans (Wanninger and Wollesen 2015). Thereby, in gastropod (and the very few investigated bivalve) larvae, a single, median, serotonin-lir flask-shaped cell often appears first, which is subsequently flanked by two or three additional flask-shaped cells (Croll et al. 1997; Kempf et al. 1997; Marois and Carew 1997a, b; Voronezhskaya et al. 2008; Kristof et al. 2016). In scaphopods, the apical organ comprises four serotonin-lir flask-shaped cells (Wanninger and Haszprunar 2003), while eight to ten cells are found in the apical organ of polyplacophorans (Friedrich et al. 2002; Voronezhskaya et al. 2002) and neomeniomorph aplacophorans (although in the latter not all show serotonin-like immunoreactivity) (Redl et al. 2014). Other identified neuroactive compounds in gastropod apical organs include leu-enkephalin (Dickinson and Croll 2003), catecholamines (Croll 2006), and various neuropeptides (FMRFamide and small cardioactive-like peptide) (Voronezhskaya et al. 2008; Ellis and Kempf 2011a, b). The emerging cerebral commissure and the anlagen of the cerebro-visceral connectives form at the base of the apical organ, while the organ itself degenerates during or prior to metamorphosis (Marois and Carew 1997a; Dickinson and Croll 2003; Wanninger and Wollesen 2015).

A comparatively low number of cells in the apical organ is commonly found in many other spiralian protostomes (Hay-Schmidt 2000, Marlow et al. 2014) and thus is likely to represent the ancestral condition for the Spiralia. However, the complex situation involving up to ten flask-like and additional peripheral cells as exemplified in polyplacophorans (and, partly, in neomeniomorph aplacophorans) is also found in the supposedly ancestral larval type of entoprocts. This, among other shared morphological features, argues in favor of a monophyletic Entoprocta + Mollusca (Tetraneuralia) (Wanninger et al. 2007; Haszprunar and Wanninger 2008, Wanninger 2009). However, since data on bivalve neurogenesis are largely lacking or remain doubtful concerning their correct interpretation (Voronezhskaya et al. 2008), the transition from a complex to a simple cellular arrangement of (serotonin-lir) cells in the apical organ within or at the base of Conchifera remains unresolved. To contribute to this question, we herein provide a detailed description of the larval nervous system in the euheterodont bivalve *Dreissena polymorpha* using antibodies against serotonin and α-tubulin. By comparison to other bivalves and mollusks, our results further stimulate the discussion concerning the ground pattern of the larval neural architecture within Bivalvia and hints towards emerging trends in the evolution of the nervous system of conchiferan mollusks on the whole.

## Materials and methods

### Animals

Sexually mature specimens of *Dreissena polymorpha* were collected in a side branch of the Danube River in Greifenstein-Altenberg, Austria. Adult specimens larger than 1 cm were gathered from stones or other hard substrates at around 30 cm depth and were transported to the laboratory. There, the shells were cleaned and the specimens were maintained individually in open plastic containers containing 100 ml filtered Danube water (FDW) at 19 °C. The water was taken from the collection site and was filtered with filter paper twice before addition to the specimens.

### Larval cultures

Spawning was induced by thermal stimulation. Thereby, the water temperature was decreased to 13 °C and then elevated to 20 °C in repetitive cycles. Spawning occurred often in the evening hours after 2–3 h of treatment. Gametes were spawned freely into the water column. Released gametes were mixed immediately and the embryos were cultivated in glass containers with 400 ml FDW at 19 °C. Water changes took place every day or every other day by gently decanting the specimens into clean culture containers with fresh FDW. Embryos and larvae of different developmental stages (gastrula, early trochophore, late trochophore, early veliger, D-shaped veliger, mid-veliger, late veliger) were relaxed by adding 7.4% MgCl_2_ and subsequently fixed in 4% paraformaldehyde (PFA) in 0.1 M phosphate buffer (PB) for 1 h at room temperature, washed in 0.1 M PB, and stored in 0.1 M PB with 0.1% NaN_3_ at 4 °C.

### Immunocytochemistry

Stages with shells (early veliger, D-shaped veliger, mid-veliger, late veliger) were decalcified in 0.5 M EGTA (pH 7.3) for 30 min, washed once with PB and unspecific binding sites were blocked overnight using 0.1 M PB (pH 7.3) with 0.5% Triton X-100 (TX) and 6% normal goat serum (NGS; Invitrogen; Molecular Probes, Eugene, OR, USA) (blockPBT) at 4 °C. All larvae were double labeled by incubating them in a mixture of mouse and rabbit primary antibodies (pAB) overnight. The following pABs were used: anti-acetylated α-tubulin (raised in mouse; Sigma; St. Louis; MO, USA) diluted 1:500 and anti-serotonin (5-HT) (raised in rabbit, Immunostar; Hudson, WI, USA) diluted 1:1000, both in blockPBT. Next, the larvae were washed for a minimum of 3 × 30 min in 0.1 M PB and 0.5% Triton X-100 (PBT) at room temperature (RT). Subsequently, the mixture of secondary antibodies (sAB) was added in a dilution of 1:200 in blockPBT to the specimens and incubated overnight in either goat anti-rabbit Alexa Flour 633 or in goat anti-rabbit Alexa Flour 568 sAB, together with goat anti-mouse Alexa Flour 488 (all Invitrogen) sAB. For the detection of cell nuclei, 1 μl HOECHST (Sigma-Aldrich; St. Louis; MO, USA) was added to the sAB solution. Additionally, HCS CellMask (Invitrogen) stainings were carried out in early trochophores to visualize cell membranes. Once again, the larvae were washed for a minimum of 3 × 30 min in PBT at RT. The stained specimens were mounted on glass slides coated with poly–L–lysine (Sigma-Aldrich; St. Louis; MO, USA) in Fluoromount-G (Southern Biotech, Birmingham, AL, USA) and stored at 4 °C for a few days prior to analysis. By omitting either the pAB, the sAB, or both, negative controls were made and yielded no fluorescent signal. Specimens were examined with a Leica SP5 II confocal laser-scanning microscope (Leica Microsystems, Wetzlar, Germany). Maximum projection images were generated and exported as TIFF files for further adjustments with LAS AF (Leica Microsystems) and IMARIS 7.3.0 (Bitplane, South Windsor, CT, USA) softwares. Adobe Photoshop CS5, Adobe Illustrator CS5, and Adobe InDesign CS6 (Adobe, San Jose, CA, USA) were used to assemble figure plates and to generate the schemes.

### Scanning electron microscopy

Specimens were prefixed in 4% PFA. After rinsing the samples in 0.1 M PB and deionized water, they were postfixed in 1% OsO_4_ for 30 min and washed again three times for 15 min each. Specimens were dehydrated in an ascending series of acetone washes (30%, 50%, 70%, 80%, 90%, 96%, 100%) for 10 min each and critical point dried in a Leica EM CPD300 dryer using CO_2_ as intermediate. They were coated with gold using an Agar B7340 (Agar Scientific, Wetzlar, Germany) sputter coater and observed with a Phillips XL20 scanning electron microscope.

## Results

### General aspects of *Dreissena polymorpha* development

**Fig. 1:**
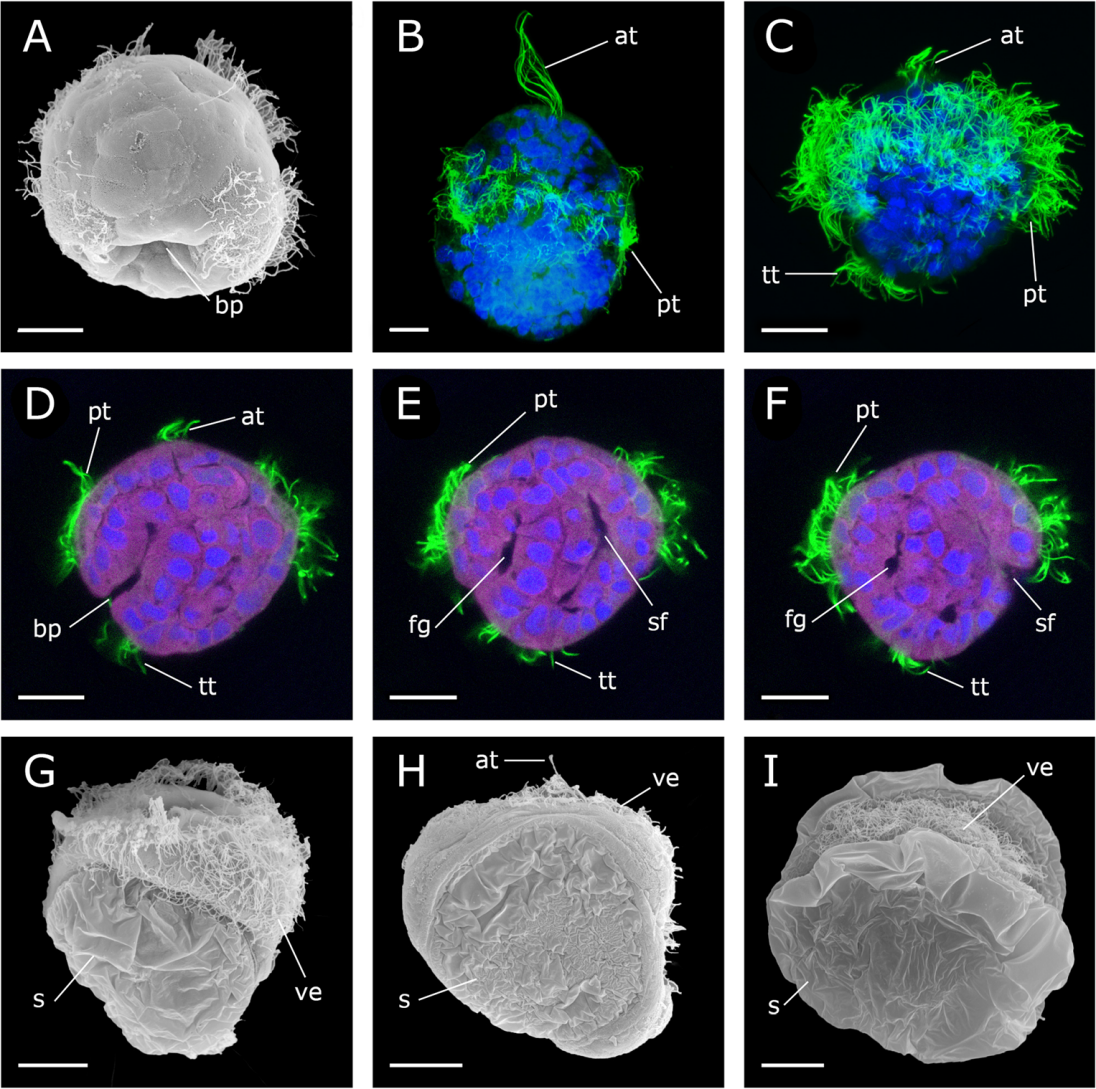
Development of *Dreissena polymorpha* from gastrula to early veliger stage. **a**, **g**, **h**, and **i** Scanning electron micrographs. **b**, **c** Confocal microscope Z-projection images. **d**, **e**, and **f** Single optical sections of **c**. Acetylated α-tubulin-lir (green), HCS CellMask (pink), and cell nuclei counter staining (blue). Apical is always up. Lateral views. Scale bars are 15 μm. **a** Ciliated gastrula stage (16 h post fertilization, hpf) with blastopore (bp) on the vegetal pole. **b** Elongated early trochophore (22 hpf) with prominent apical tuft (at) and prototroch (pt). **c** Early-trochophore (23 hpf) with apical tuft (at), prototroch (pt), and telotroch (tt). **d** Early trochophore (23 hpf). **e**, **f** Early trochophore (23 hpf) in different optical planes with foregut (fg) and shell field (sf) invagination. **g** Early veliger (39 hpf) with embryonic shell (s) and expanded velum (ve). **h** 46 hpf old veliger. **i** Late veliger larva (188 hpf)

Development was monitored under a constant temperature of 19 °C. Between 14 and 18 h post fertilization (hpf), the animals developed into ciliated, free-swimming gastrulae, which are characterized by the presence of two distinct depressions, the blastopore and the shell field invagination (Fig. 1a, e). At about 20 hpf, an elongated early trochophore develops that measures 80–90 μm along the anterior-posterior axis. It has an equatorial band of ciliated cells, the prototroch, and an apical tuft (Fig. 1b). The telotroch, a patch of ciliated cells at the posterior larval pole, appears slightly later, in about 24 hpf trochophores (Fig. 1c). At this stage, the invagination of the shell field and the blastopore are visible (Fig. 1d–f). Between 39 and 50 hpf, the larva develops into an early veliger with a digestive system. The prototroch expands and forms the velum. The shell has not yet developed completely (Fig. 1g, h). Around the second day of development, the shell enlarges rapidly and soon envelops the entire larva, giving it a D-shape appearance. These larvae (90–100 μm long) are referred to as D-stage veligers. By 3–4 days post fertilization (dpf), the larva is in the mid-veliger stage and loses its prominent apical tuft. After 5–6 days, the larva reaches the late veliger stage. The D-shape begins to change and an umbo forms on the dorsal side (Fig. 1i). Compared to the D-stage veliger, the late veliger larvae have not increased in size, still measuring 90–100 μm. During the late veliger stage (veliconcha), a continuous shrinking of the velum in size appears. The eighth day marked the day of settlement and the onset of metamorphosis in the present study.

### Neurogenesis of the serotonin- and α-tubulin-lir nervous system

**Fig. 2:**
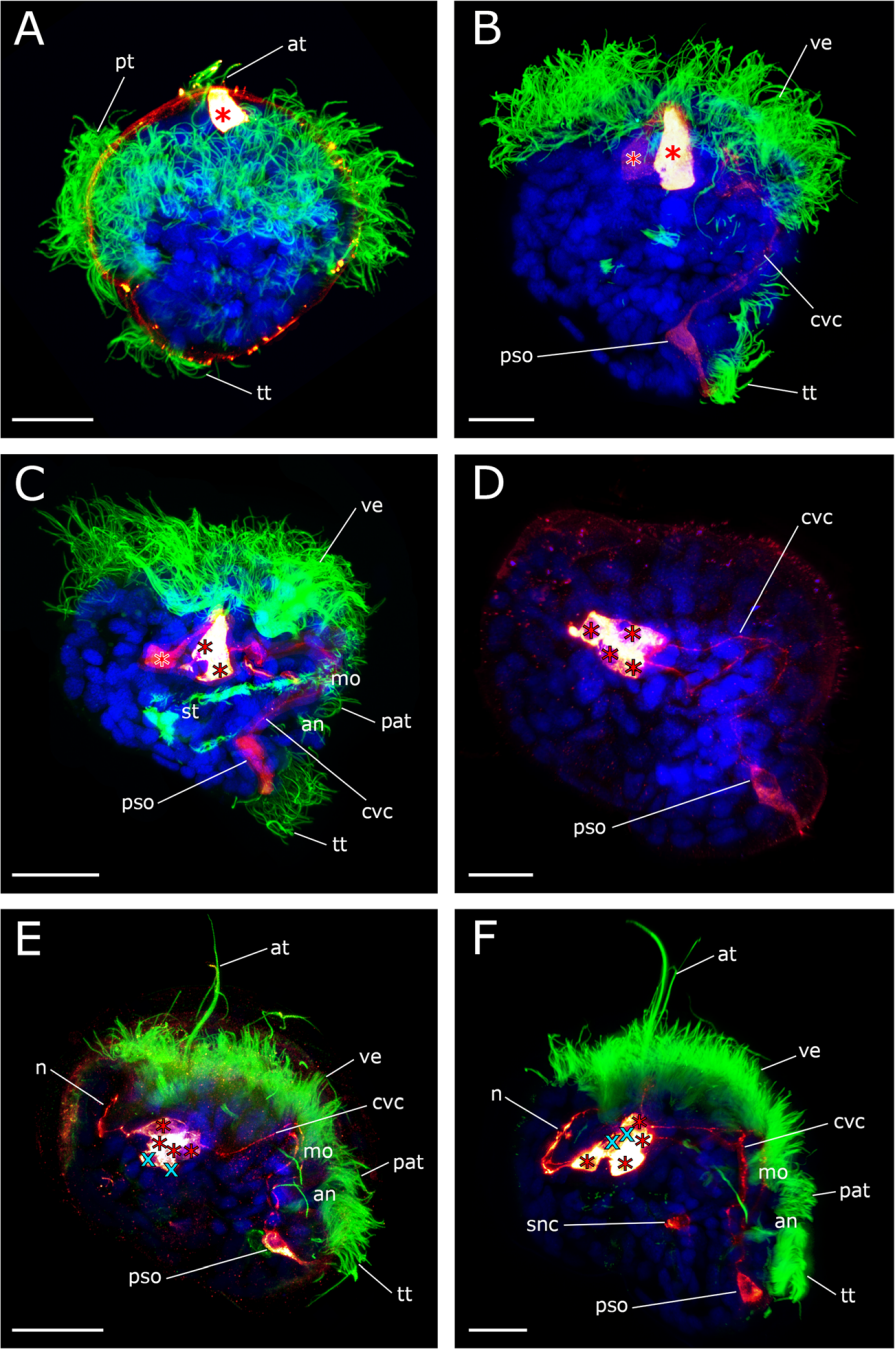
Development of the serotonin-lir nervous system in *Dreissena polymorpha* from trochophore to early veliger stage. Serotonin-lir (bright-yellow to dark-red), acetylated α-tubulin-lir (green), and cell nuclei counter staining (blue). All images are in lateral view and apical is always up. Scale bars are 15 μm. **a** Trochophore larva (23 hpf). First serotonin-lir flask-shaped cell (red asterisk) at the apical pole. (at) apical tuft, (pt) prototroch, (tt) telotroch. **b** Early veliger larva (39 hpf). Two flask-shaped serotonin-lir cells (red asterisks) in the apical organ underlying the velum (ve). Postero-ventrally, the posterior larval sensory organ (pso) develops. Faintly labeled paired cerebro-visceral connectives (cvc) connect the posterior larval sensory organ (pso) to the apical organ. **c** Early veliger larva (50 hpf). Three serotonin-lir cells (red asterisks) form the apical organ. (an) anus, (mo) mouth opening, (pat) pre-anal tuft. (st) stomach. **d** D-shaped veliger larva (62 hpf). Four flask-shaped serotonin-lir cells (red asterisks) constitute the apical organ. **e**, **f** D-shaped veliger larva (69 hpf). Underlying the apical organ (red asterisks), two roundish non-flask-shaped cells (turquoise x) appear. These cells form the anlage of the future cerebral ganglion (cg). Neurites (n) from the apical organ project into the velum (ve) and anteriorly innervate the apical tuft (at). One additional unpaired serotonin-lir stomatogastric nerve cell (snc) appears ventrally to the stomach

**Fig. 3:**
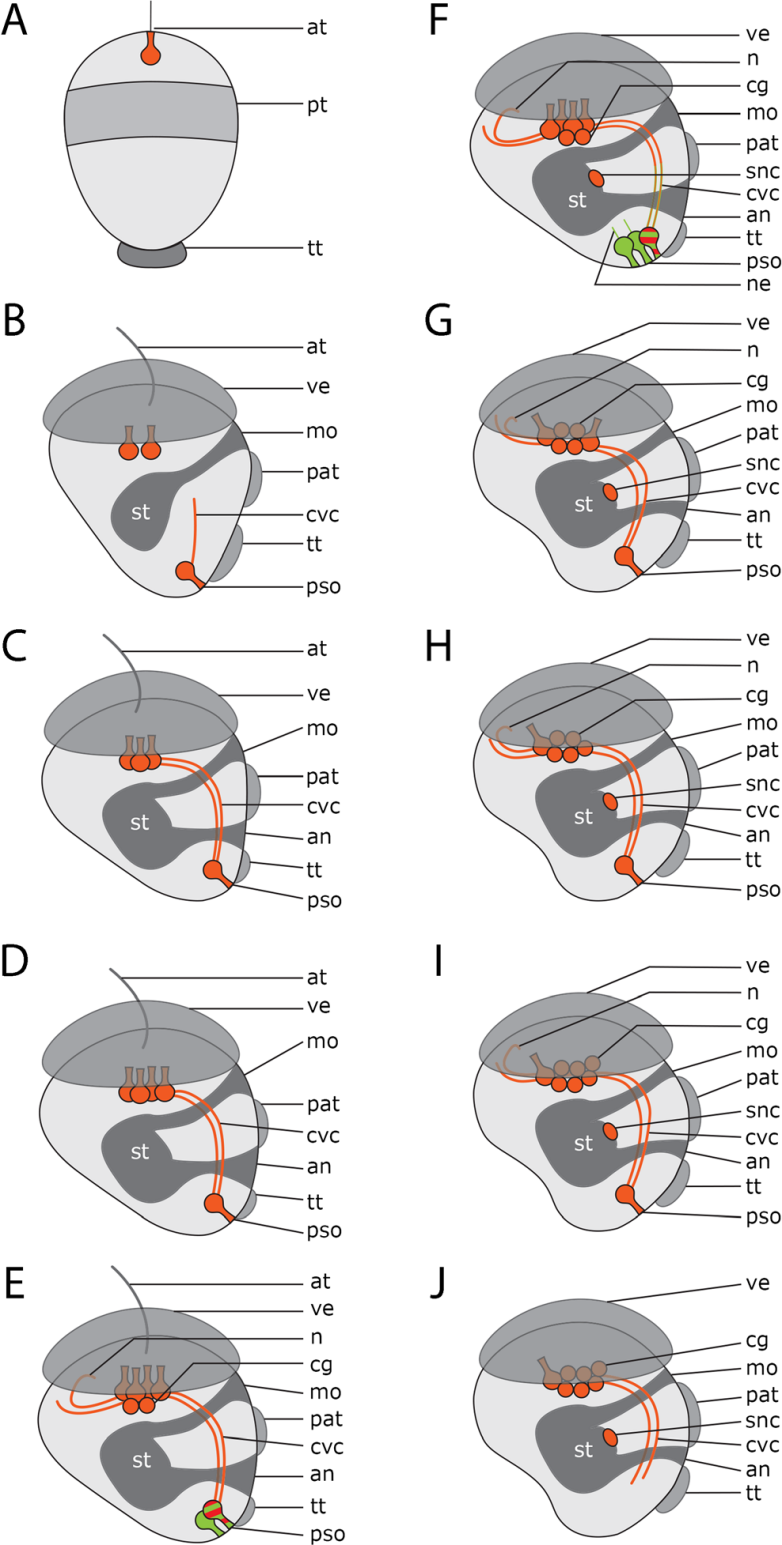
Schematic drawings of serotonin-lir neurogenesis in *Dreissena polymorpha*. Serotonin-lir cells are red and acetylated α-tubulin-lir cells are green. All images in lateral view with apical up. (an) anus, (at) apical tuft, (cg) anlage of cerebral ganglion, (mo) mouth opening, (n) neurite projecting into the velum, (ne) neurites projecting from the posterior larval sense organ, (pat) pre-anal tuft, (pso) posterior larval sensory organ, (pt) prototroch, (snc) stomatogastric nerve cell, (st) stomach, (tt) telotroch, (ve) velum, (cvc) cerebro-visceral connectives. **a** The first serotonin-lir flask-shaped apical cell appears in the trochophore stage. **b** In the early veliger, two serotonin-lir flask-shaped cells are present in the apical organ, as is the first-formed serotonin-lir flask-shaped cell of the posterior larval sensory organ and the anlage of the ventral neurites. **c** Slightly later three flask-shaped cells are present in the apical organ. Note the paired cerebro-visceral connectives which connect the posterior larval sensory organ to the apical organ of the larva. **d** At the D-shaped veliger stage, the apical organ consists of four flask-shaped cells. **e** Slightly later two roundish, non-sensory cells appear which underlie the apical organ and form the anlage of the future cerebral ganglion. Neurites project into the velum. A second flask-shaped cell in the posterior larval sensory organ appears. This cell shows only α-tubulin-lir, while the first-formed cell shows immunoreactivity against serotonin and α-tubulin. **f** In the mid-veliger stage, a third flask-shaped cell in the posterior larval sensory organ appears. It shows only α-tubulin-lir*.* Both of the α-tubulin-lir cells send short neurites in anterior direction. Note the stomatogastric nerve cell. **g** Slightly later the two α-tubulin-lir cells in the posterior larval sensory organ disappear and only the first-formed serotonin-lir flask-shaped cell remains. In the apical organ, two flask-shaped cells have disappeared. It now comprises two flask-shaped cells and four roundish, non-flask-shaped cells, which are the anlage of the future cerebral ganglion. **h** Only one flask-shaped cell is left in the apical organ. Five roundish cells form the anlage of the future cerebral ganglion. **i** In the late veliger six roundish, non-flask-shaped cells constitute the anlage of the future cerebral ganglion. **j** The immunoreactive cells of the posterior larval sensory organ have disappeared

The first serotonin-lir flask-shaped, cilia-bearing cell appears in the trochophore stage (20–24 hpf) at the anterior pole of the larvae (Figs. 2a and 3a). It is the first cell of the future apical organ. Shortly thereafter, at the early veliger stage (39hpf), a second serotonin-lir flask-shaped cell appears adjacent to the first one. At the same time, the first neurites that project posteriorly from the apical cells are visible and constitute the anlagen of the future cerebro-visceral connectives (Figs. 2b and 3b). One additional serotonin-lir flask-shaped cell with short immunopositive neurites that project anteriorly emerges postero-ventrally (Figs. 2b and 3b). This cell shows only weak serotonin-lir and comprises the first-formed cell of the posterior larval sensory organ.

Slightly later, in the early veliger stage (50 hpf), at least two additional serotonin-lir flask-shaped cells appear one after another in the anterior region and together form the larval apical organ (Figs. 2c and 3c, d). The cluster of cells of the apical organ seems to become more compact at this stage. Simultaneously, with the formation of the larval apical organ, the neurites projecting posteriorly from the apical organ and anteriorly from the posterior larval sensory organ are interconnected and constitute the paired cerebro-visceral connectives (Figs. 2d and 3c, d). No further changes in the position of the paired cerebro-visceral connectives were observed throughout subsequent larval development.

By 2–3 dpf (i.e., in the D-shaped veliger), the cluster of the four flask-shaped apical cells becomes more compact and appears to have subsided into the larval body. Below the apical organ, two roundish non-flask-shaped serotonin-lir cells appear. Due to the tight clustering of these cells, it is not possible to elucidate if these cells are uni- or bipolar. At this time, the first serotonin-lir neurites that project from the apical organ dorsally into the velum are formed (Figs. 2e and 3e).

**Fig. 4:**
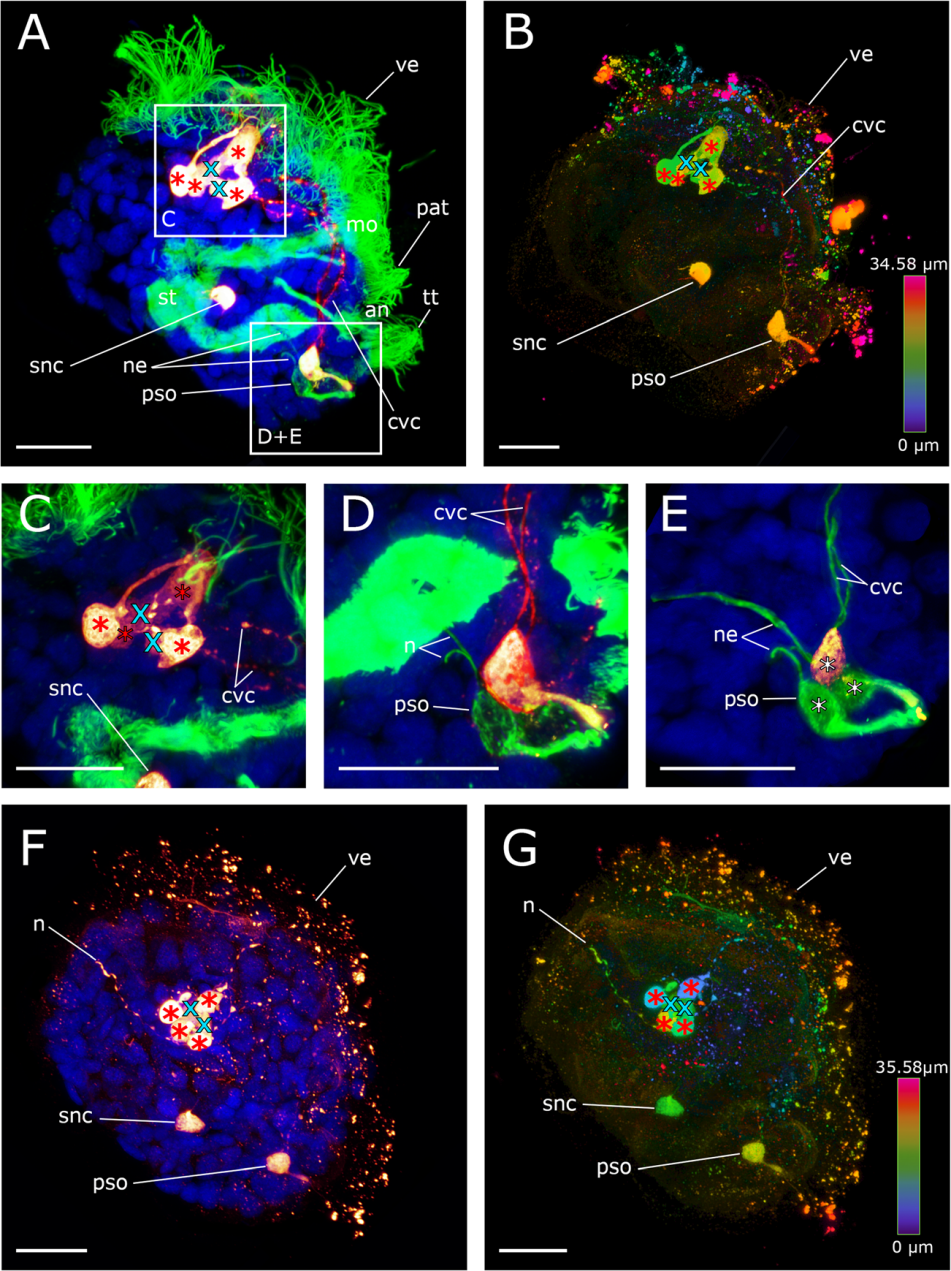
Components of the serotonin-lir nervous system in the mid-veliger stage of *Dreissena polymorpha*. Serotonin-lir (bright yellow to dark red), acetylated α-tubulin-lir (green), and cell nuclei counter staining (blue). **c**, **d**, **e** Details of **a**. All images are in lateral view and apical is always up. Scale bars are 15 μm. **a** Overview of major neural components including four flask-shaped serotonin-lir cells (red asterisks) that form the apical organ. Neurites (n) project dorsally into the velum (ve). The anlage of the cerebral ganglion (turquoise x) is located underneath the apical organ. Cerebro-visceral connectives (cvc) connect the posterior larval sensory organ (pso) with the apical organ (ao). **b** Same individual as in **a** but color-coded for depth. **c** Detail of the apical organ (red asterisks) and the anlage of the cerebral ganglion (turquoise x). **d** Posterior larval sensory organ (pso) with first-formed cell and anteriorly projecting cerebro-visceral connectives (cvc) showing serotonin-lir components. Flask-shaped cell with neurites (n) projecting medially showing α-tubulin-lir components. **e** Posterior larval sensory organ (pso) consisting of three flask-shaped cells (white asterisks) with expanding neurites (ne, vn), which are all α-tubulin-lir. Only the first-formed cell with expanding cerebro-visceral connectives (cvc) shows additional serotonin-lir. (an) anus, (pat) pre-anal tuft, (mo) mouth, (snc) stomatogastric nerve cell, (st) stomach, (tt) telotroch. **f** Larva showing a neurite (n) projecting from the apical organ into the velum. **g** Same individual as in **f** but color-coded for depth

**Fig. 5:**
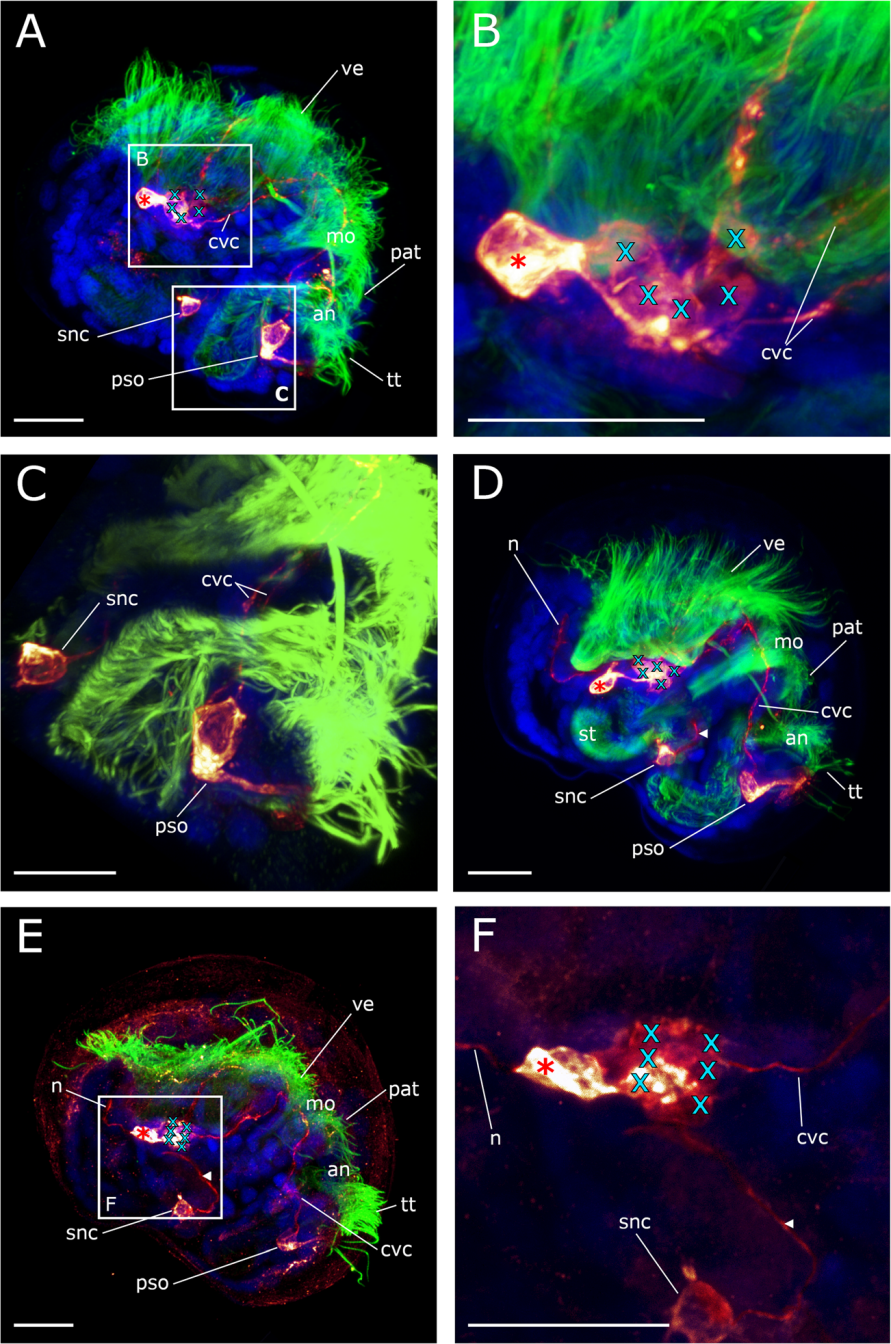
Development of the serotonin-lir nervous system in *Dreissena polymorpha* from mid- to late veliger stage. Serotonin-lir (bright yellow to dark red), acetylated α-tubulin-lir (green), and cell nuclei counter staining (blue). **b**, **c** Details of **a**. **f** Detail of **e**. All images are in lateral view and apical is always up. Scale bars are 15 μm. **a** Mid-veliger larva (114 hpf). One remaining flask-shaped cell of the larval apical organ (red asterisk) underlain by the anlage of the cerebral ganglion which contains five roundish non-flask-shaped cells (turquoise x). Paired cerebro-visceral connectives (cvc) project from the anlage of the cerebral ganglion to the posterior larval sensor organ (pso). (an) anus, (mo) mouth opening, (tt) telotroch **b** Detail of the remaining flask-shaped cell of the apical organ (red asterisk) and the anlage of the cerebral ganglion (turquoise x). **c** Detail of the posterior larval sensory organ (pso) and the unpaired stomatogastric nerve cell (snc). **d** Mid-veliger larva (114 hpf). Neurites (n) project from the remaining apical cell (red asterisk) dorsally into the velum (ve). A neurite (arrowhead) runs from the stomatogastric nerve cell (snc) in anterior direction. (st) stomach, (cvc) cerebro-visceral connectives. **e** Late veliger larva (165 hpf). The anlage of the cerebral ganglion now consists of six non-flask-shaped cells (turquoise x). From the unpaired stomatogastric nerve cell (snc), a neurite (arrowhead) projects into the anlage of the cerebral ganglion. **f** Detail of **e**. (pat) pre-anal tuft

**Fig. 6:**
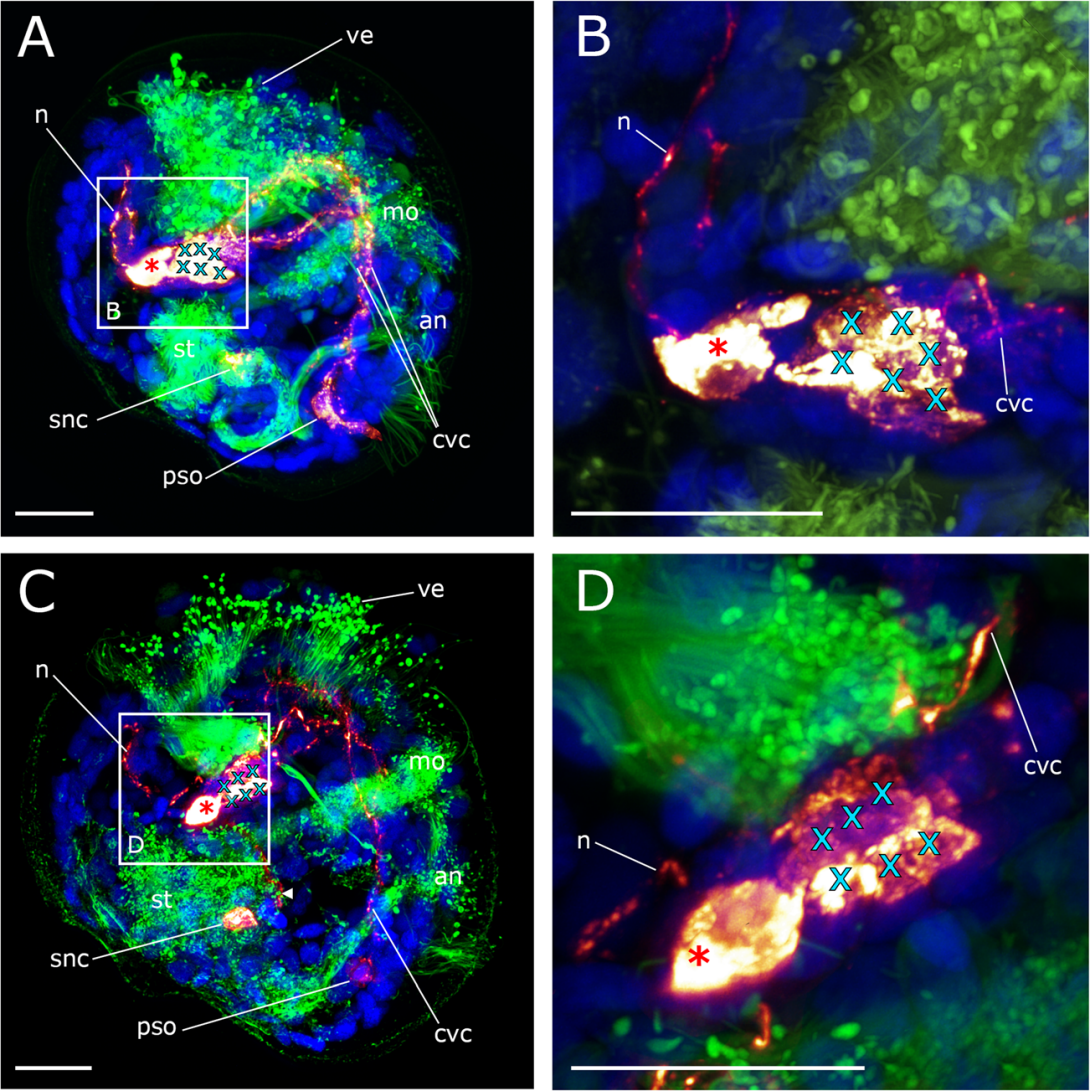
Components of the serotonin-lir nervous system in the late veliger larva of *Dreissena polymorpha*. Serotonin-lir (bright yellow to dark red), acetylated α-tubulin-lir (green), and cell nuclei counter staining (blue). All images are in lateral view and apical is always up. Scale bars are 15 μm. **a** One flask-shaped serotonin-lir cell (red asterisk) remains of the apical organ and a neurite (n) projects dorsally into the velum (ve). The anlage of the future cerebral ganglion consists of six round, non-flask-shaped cells (turquoise x). (an) anus, (mo) mouth opening, (st) stomach. **b** Detail of **a**. Paired cerebro-visceral connectives (cvc) project from the anlage of the cerebral ganglion to the disappearing posterior larval sensory organ (pso). **c** Late veliger larva (188 hpf). Neurites (n) project from the apical organ (red asterisk) dorsally into the reduced velum (ve) and into a posterior direction (arrowhead). (snc) stomatogastric nerve cell. **d** Detail of **c**. From the anlage of the cerebral ganglion, consisting of six non-flask-shaped cells (turquoise x), paired cerebro-visceral connectives (cvc) project to the posterior pole of the larva where the posterior larval sensory organ (pso) starts to disappear

In the mid-veliger stage (95 hpf), one single serotonin-lir stomatogastric nerve cell appears ventrally to the stomach of the larva (Figs. 2f and 3f). About that time, the number of flask-shaped cells in the posterior larval sensory organ increases to three (Figs. 3f and 4a). All three cells are immunopositive against α-tubulin but only the first-formed cell, from which the cerebro-visceral connectives originate, is also immunoreactive to serotonin (Figs. 2b and 3f). The cells of the posterior larval sensory organ send α-tubulin-lir neurites in an anterior direction but the termination of these neurites could not unambiguously be followed and were only found in this stage (Figs. 3f and 4d). In the mid-veliger (114 hpf), the flask-shaped cells of the apical organ start to shrink in size and lose their typical flask-shaped appearance (Fig. 4). At this stage, only one serotonin-lir flask-shaped cell can be identified within the apical organ (Fig. 3h and 5a). The number of non-flask-shaped, round serotonin-lir cells increases to five and subsequently, in the late veliger stage (165 hpf), to six, that all form a dense cluster (Figs. 3h, i; 5; and 6). The cerebro-visceral connectives previously connected to the apical organ now seem to originate from this cluster of round serotonin-lir cells. A short serotonin-lir neurite projects in anterior direction from the stomatogastric nerve cell adjacent to the apical organ (Figs. 5d–f and 6c, arrowheads). At this stage, immunoreactivity in the posterior larval sensory organ decreases significantly in comparison to the previous stage (Figs. 3i, j and 6c).

## Discussion

### Comparative aspects of bivalve serotonin-lir neurogenesis

**Fig. 7:**
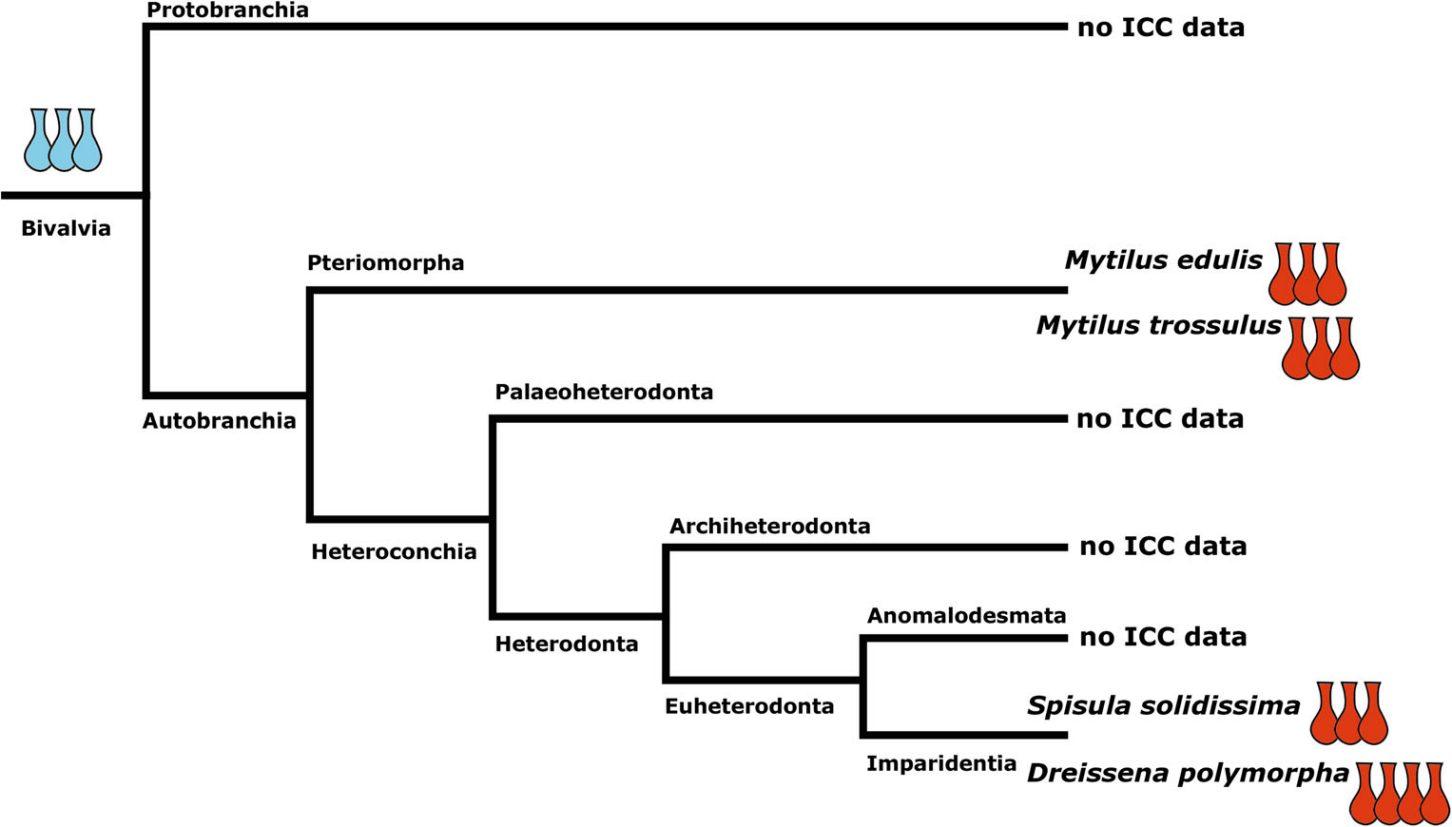
Suggested ground patterns based on available data for numbers of serotonin-lir apical flask-shaped cells within bivalve apical organs. For further assessment, data on crucial clades, in particular Palaeoheterodonta and Protobranchia, are vital. Phylogeny of major bivalve lineages based on González et al. (2015). Red flask-shaped cells represent the cell count of respective serotonin-lir cells in the apical organ of studied species. Blue cells represent the hypothetical ground pattern. Within Heterodonta, *Spisula solidissima* shows three flask-shaped cells, while *Dreissena polymorpha* shows four. Within Pteriomorpha, *Mytilus edulis* shows three flask-shaped cells. *Mytilus trossulus* has five cells, but only 3 appear to be flask-shaped. Data on the larval serotonin-lir nervous system on the basally branching Protobranchia are still lacking. Taken together, the data currently available suggest a ground pattern of three flask-shaped cells for the larva of the last common bivalve ancestor but additional data for various lineages are needed to further solidify this assumption

A recent phylogenomic analysis suggests that Bivalvia comprises five major clades, Protobranchia, Pteriomorpha, Palaeoheterodonta, Archiheterodonta, and Euheterodonta (Fig. 7). *Dreissena polymorpha* resides within Euheterodonta that, together with Archiheterodonta, forms the Heterodonta (González et al. 2015). Immunocytochemical data dealing with various aspects of bivalve early neurogenesis are available only for pteriomorphs (e.g., Croll et al. 1997; Voronezhskaya et al. 2008; Ellis and Kempf 2011a, 2011b; Audino et al. 2015) and euheterodonts (e.g., Kreiling et al. 2001; Altenöder and Haszprunar 2008), while for Protobranchia, the basal-most branching taxon and the sister group to all other bivalve subgroups, only one detailed study on larval neuroanatomy employing electron microscopy methods has been published (Zardus and Morse 1998). This lack of data significantly hampers ground pattern reconstruction for bivalve larval nervous systems. However, certain evolutionary hypotheses on putative ancestral features of bivalve larval anatomy are beginning to emerge, which are discussed in the following with respect to the most important neural subsets hitherto identified (see also Fig. 7).

#### Apical organ

In all investigated larval bivalves including *Dreissena polymorpha*, an apical organ was found from early developmental stages onwards. *D. polymorpha* larvae have an apical organ that contains four serotonin-lir flask-shaped cells, while the apical organ of *Spisula solidissima* larvae consists of three serotonin-lir flask-shaped cells (Kreiling et al. 2001). In the apical organ of *Mytilus trossulus*, five serotonin-lir cells are present, of which three were described as flask-shaped (Voronezhskaya et al. 2008). This number is consistent with its congener, *Mytilus edulis* (Croll et al. 1997). Accordingly, three to four serotonin-lir flask-shaped cells are found in pteriomorphs and euheterodonts.

There is a noticeable degeneration of the apical organ with the loss of the apical tuft in *Dreissena polymorpha* towards metamorphosis. The immunopositive cells lose their typical flask-shaped pattern and appear to form a more complex cluster with six additional serotonin-lir non-flask-shaped cells underlying the apical organ. In *M. trossulus*, no such additional cells were found. These cells retain their position in early post-metamorphic stages in this species and may constitute the early anlagen of the cerebral ganglia (Voronezhskaya et al. 2008). In *Spisula solidissima*, additional serotonin-lir cells were not found in the apical region (Kreiling et al. 2001). To assess the future fate of the six additional serotonin-lir non-flask-shaped cells in *Dreissena polymorpha*, further investigations of later developmental stages are required.

#### Cerebral ganglia

*Dreissena polymorpha* larvae show six serotonin-lir non-flask-shaped cells in the apical region outside the apical organ whose position suggests that they constitute parts of the developing cerebral ganglia. In *Mytilus trossulus*, the five serotonin-lir cells of the apical organ were described to lose their cilia and flask-like shape and are incorporated in the developing cerebral ganglia (Voronezhskaya et al. 2008). If true, this would be the first case of a direct ontogenetic continuation and integration of sensory cells of a larval apical organ into an adult central nervous system of any mollusk and probably any lophotrochozoan. However, since other aspects presented in this study (Voronezhskaya et al. 2008) remain doubtful (see below), these results should be treated with utmost care.

#### Velum-innervating serotonin-lir neurites

In contrast to most other molluscan larvae, *Dreissena polypmorpha* does not show a serotonin-lir neural plexus or nerve ring underlying the prototroch/velum. However, the velum in *Dreissena polymorpha* is innervated by a serotonin-lir neurite that emerges from the apical organ and projects into the dorsal part of the velum. Such a serotonin-lir innervation of the velum is lacking in *Spisula solidissima* (Kreiling et al. 2001) and in *Mytilus edulis* (Croll et al. 1997). However, short serotonin-lir neurites that project dorsally towards the velum were found in *Mytilus trossulus* (Voronezhskaya et al. 2008)*.* In *Crassostrea virginica* there is a similar extension from the apical organ into the dorsal portion of the velum, yet only catecholamine-lir has been described from this neurite (Ellis and Kempf 2011a, b). Thus, a dorsal serotonin-lir innervation of the velum is shared by some of the bivalve larvae investigated to date and this may constitute a conserved bivalve feature. However, additional data particularly from representatives of the supposedly earliest branching bivalves, the protobranchs, are needed to further assess this issue.

#### Cerebro-visceral connectives and posterior larval sensory organ

*Dreissena polymorpha* shows one pair of serotonin-lir neurites that project postero-ventrally. These neurites constitute the paired cerebro-visceral connectives and terminate in the so-called “posterior larval sensory organ.” In *Mytilus trossulus*, similar, ventrally projecting serotonin-lir fibers that emerge from the apical organ were observed, but their target sites could not be determined (Voronezhskaya et al. 2008). The FMRFamide-lir connectives in *M. trossulus* were described to form a continuous neurite bundle that interconnects the cerebral, pedal, and visceral ganglia, a situation unlike that in any other mollusk and incompatible with the typical molluscan tetraneural condition. Accordingly, a specific designation of these neurite bundles as cerebro-visceral or cerebro-pedal connective is impossible based on the data presented, calling for reinvestigation of neurogenesis in this species (cf. Voronezhskaya et al. 2008). In *Spisula solidissima*, a serotonin-lir process emerges from the apical organ and extends into the visceral ganglion (Kreiling et al. 2001). With respect to the position within the larval body and developmental stage, these serotonin-lir fibers and the process in *S. solidissima* are topographically similar to the cerebro-visceral connectives of *D. polymorpha*.

The posterior larval sensory organ in *Dreissena polymorpha* contains three flask-shaped ciliated cells and this is the first study that reports immunoreactivity in cells of this bivalve-specific organ. The ontogenetically first-formed cell shows co-localization of serotonin and α-tubulin, whereas the other two cells exhibit α-tubulin-lir only. Due to the postero-ventral position of these cells within the larval body and the presence of flask-shaped sensory cells, it is considered homologous to the so-called “ciliated post-anal organ” described in the lecithotrophic test cell larva of the protobranch *Acila castrensis* using transmission electron microscopy (Zardus and Morse 1998). Although such an organ has not been described for any other bivalve so far, its presence in a protobranch representative makes it tempting to suggest that it is part of the bivalve larval ground pattern, but further studies on additional taxa are needed to assess this assumption.

#### Stomatogastric nerve cell

*Dreissena polymorpha* shows one serotonin-lir cell in a median position ventrally of the stomach. The cell appears in the D-shaped veliger stage slightly later than the cells of the proposed anlage of the cerebral ganglion. At this stage, the larva has a fully developed digestive tract. In the mid-veliger stage, neurites extend from the serotonin-lir cell near the stomach in an anterior direction, but they never interconnect with any other neural structure. In the late veliger stage, the serotonin-lir signal of the cell disappears. According to the location and origin of formation, the cell is likely to serve the digestive tract and is thus called “stomatogastric nerve cell” herein. Interestingly, no comparable serotonin-lir cell has been described in any other bivalve larva so far. However, comparable cells are known from *Mytilus edulis* pediveliger and *Mytilus trossulus* veliger larvae, which exhibit catecholamine-lir and were described as “abdominal ganglia” (Croll et al. 1997; Voronezhskaya et al. 2008). In *Crassostrea virginica*, a small cardioactive peptide-like innervation along the larval esophagus is present but seems to form a complex network with esophageal neurons (Ellis and Kempf 2011b). Further investigations are needed for more detailed functional assessments and to determine the future fate of this stomatogastric nerve cell after metamorphosis.

#### Pedal and visceral ganglia

In *Dreissena polymorpha* pedal or visceral ganglia exhibiting serotonin-lir or tubulin-lir could not be detected in larvae prior to the advanced veliger stage. All other studies on bivalve larvae except for *Spisula solidissima* showed immunoreactive pedal ganglia. However, in this latter species, serotonin-lir cells are present in the visceral ganglia (Kreiling et al. 2001). The pedal ganglia in *Mytilus edulis* and *Mytilus trossulus* show catecholamine-lir cells, and *M. trossulus* shows additional FMRFamide-lir cells. There is no immunoreactivity in the visceral ganglia of the larva of *M. edulis*, while in *M. trossulus* each visceral ganglion exhibits catecholamine-lir and two to three FMRFamide-lir cells (Croll et al. 1997; Voronezhskaya et al. 2008). In the D-shaped veliger stage of *Crassostrea virginica*, the small cardioactive peptide-like immunoreactivity is limited to the apical region of the larva. Only in the late veliger stage a maximum of one small cardioactive peptide-like cell is present in each pedal ganglion and three to four in the visceral ganglia (Ellis and Kempf 2011b).

In summary, the majority of bivalve larvae investigated to date show a serotonin-lir apical organ with three to five flask-shaped cells. Furthermore, serotonin-lir components are present in some representatives that may contribute to the future cerebral ganglion. A serotonin-lir dorsal innervation of the velum as well as cerebro-visceral connectives that appear early in development are common in bivalve larvae. All these shared features are likely to have been present in the larva of the last common bivalve ancestor, although data on the putatively basally branching protobranchs are needed to further test this hypothesis. The posterior larval sensory organ has so far only been described in *Dreissena polymorpha* and the protobranch *Acila castrensis* and additional bivalve representatives should be specifically tested for its presence during development.

### Comparative aspects of the serotonin-lir apical organ within Mollusca

According to recent phylogenomic analyses, Mollusca is subdivided into two monophyletic taxa, Aculifera and Conchifera (Kocot et al. 2011; Smith et al. 2011). Both studies agree that within Aculifera, Polyplacophora forms the sister group to the two aplacophoran clades, Chaetodermomorpha and Neomeniomorpha. However, the phylogenetic interrelationships of the conchiferan taxa (Monoplacophora, Bivalvia, Gastropoda, Scaphopoda, Cephalopoda) remain largely unresolved.

In relatively basally branching gastropods, the apical organ constitutes a simple structure with only two flask-shaped serotonin-lir cells in the vetigastropod *Haliotis kamtschatka* (Page 2006), while patellogastropods have a more complex cellular composition (three flask-shaped, one median and two lateral serotonin-lir cells in *Tectura scutum*; one median, two lateral, and two additionally round serotonin-lir cells in *Lottia* cf. *kogamogai*; Page 2002, Kristof et al. 2016). The neritimorph *Nerita melanotragus* shows an apical organ with four serotonin-lir non-flask-shaped cells and multiple α-tubulin-lir cells that form so-called “sensory cups” (Page and Kempf 2009). In contrast to all other pelagic gastropod larvae investigated to date, *N*. *melanotragus* lacks serotonin-lir flask-shaped cells within the apical organ (Page and Kempf 2009).

The complex situation found in *Lottia* cf. *kogamogai* is shared with more derived gastropods such as opisthobranchs, caenogastropods, and nudibranchs. In the opisthobranch gastropod *Aplysia californica*, the apical organ contains five serotonin-lir cells. Three of them are flask-shaped, one medially and two of them laterally positioned, and two are non-flask-shaped, round cells (Marois and Carew 1997a; Dickinson et al. 2000; Kempf and Page 2005; Wollesen et al. 2007). Larvae of the caenogastropods *Euspira lewisii, Lacuna vincta*, *Trichotropis cancellata*, *Amphissa versicolor*, and *Ilyanassa obsoleta* have serotonin-lir cells within the apical organ and neurites within the velum (Page and Parries 2000; Dickinson and Croll 2003). *E. lewisii* and *A. versicolor* both show an identical arrangement and number of five serotonin-lir cells within the apical organ as mentioned above for *A. californica*, while in *L. vincta*, the median flask-shaped cell is lacking. In *T. cancellata*, there are three serotonin-lir cells, one median flask-shaped, and two lateral non-flask-shaped cells. In contrast, *A. versicolor* and *I. obsoleta* show an additional non-flask-shaped cell on the right side and thus the apical organ is composed of six serotonin-lir cells in total (Page and Parries 2000; Dickinson and Croll 2003). Accordingly, in caenogastropod larvae, the number of serotonin-lir cells ranges from three to six, but one pair of non-flask-shaped serotonin-lir lateral cells is common to all of them (Page and Parries 2000).

In the nudibranch gastropods *Aeolidiella stephanieae* (Kristof and Klussmann-Kolb 2010), *Phestilla sibogae* (Croll 2006) and others (Kempf et al. 1997) five serotonin-lir cells are part of the apical organ. Such a pattern consists of a median flask-shaped cell, a pair of lateral flask-shaped cells, and two non-flask-shaped, round cells. Accordingly, in opisthobranchs, nudibranchs, and caenogastropods, an apical organ consisting of a median flask-shaped cell, a pair of lateral flask-shaped cells, and two non-flask-shaped, round cells is common. In comparison to the larvae of these later branching gastropods, the larvae of basally branching patello- and vetigastropods show a considerably lower number of flask-shaped cells in the apical organ, usually three (see Kristof et al. 2016 for recent summary).

In the scaphopod *Antalis entalis*, neurogenesis starts with the apical organ, which consists of four serotonin-lir flask-shaped cells, a number similar to bivalves and basal gastropods (Wanninger and Haszprunar 2003). Accordingly, the data currently available for Conchifera suggests that a comparatively simple apical organ of few (three to five) serotonin-lir flask cells was part of the ground pattern of the larval nervous system of Conchifera.

In aculiferans, the situation is strikingly different to that of the conchiferans. In polyplacophorans, the number of cells in the apical organ is significantly higher than in conchiferan larvae and contains eight to ten serotonin-lir flask-shaped cells which are surrounded by numerous peripheral cells (Friedrich et al. 2002; Haszprunar et al. 2002; Voronezhskaya et al. 2002). In the neomeniomorphs, neurogenesis starts simultaneously from the apical and abapical pole with an apical organ and a posterior neurogenic domain (Redl et al. 2014). Here, only two of about ten flask-shaped cells contain serotonin, while the presence of the others could only be revealed by α-tubulin immunoreactivity (Redl et al. 2014). Peripheral cells comparable to those found in polyplacophoran larvae were not found. In the light of recent comparative developmental studies that suggest a polyplacophoran-like last common aculiferan ancestor (Scherholz et al. 2013, 2015), it is tempting to speculate that a complex apical organ is also ancestral for Aculifera. This scenario would gain further support if independent (molecular-based) confirmation of the Tetraneuralia concept (i.e., a monophyletic Mollusca + Entoprocta) would become available, since the supposed ancestral entoproct larval type likewise exhibits a complex apical organ (Wanninger et al. 2007). However, admittedly, data on key taxa are still lacking, even within Mollusca (e.g., on monoplacophorans or protobranch bivalves), as are reliable internal phylogenies for Mollusca and the entire Spiralia, thus hampering solid ground pattern reconstructions for various nodes within the lophotrochozoan tree of life.
